# Dietary Supplements Questioned in the Polish Notification Procedure upon the Basis of Data from the National Register of Functional Foods and the European System of the RASFF

**DOI:** 10.3390/ijerph19138161

**Published:** 2022-07-03

**Authors:** Kacper Wróbel, Anna Justyna Milewska, Michał Marczak, Remigiusz Kozłowski

**Affiliations:** 1 Department of Management and Logistics in Healthcare, Medical University of Lodz, 90-131 Lodz, Poland; michal.marczak@umed.lodz.pl; 2 Department of Statistics and Medical Informatics, Medical University of Bialystok, 15-089 Bialystok, Poland; anna.milewska@umb.edu.pl; 3 Centre for Security Technologies in Logistics, Faculty of Management, University of Lodz, 90-237 Lodz, Poland; remigiusz.kozlowski@wz.uni.lodz.pl

**Keywords:** dietary supplements, RASFF, GIS, notification

## Abstract

Dietary supplements (DS) in the countries of the European Union falls within the scope of the food law. DS may, however, contain substances that are simultaneously applied in medicinal products as defined in the pharmaceutical law. The presence of such ingredients may cause problems with the product qualification. The phenomenon of applying such borderline ingredients in dietary supplements may require additional regulations, and ensuring them may be problematic. We conducted an analysis aiming to identify dishonest market practices resorted to by the producers and distributors of non-conforming dietary supplements. We examined mostly questioned DS and compared them with data from the RASFF system and registers of medicinal substances and pharmaceutical entities. The results show that some operators tend to re-notify the same products in response to the initiation of official control procedures. Products in the form of capsules or powders were the most common re-notifications within the 50–100 days. Based on the data obtained, it can be concluded that some entities are obliged to document the safety of the product or its compliance with the regulations, use the imperfection of the notification procedure, and re-notify the questioned product in order to keep it on the market despite potential non-compliance.

## 1. Introduction

Dietary supplements (DS) production and trading in the countries of the European Union falls within the scope of a number of the legal regulations generally referred to as the “food law”. Particular laws regulate actions conducted at the consecutive stages of the food chain to a different degree at the national and European Union levels [1].

DS may, however, contain substances that are simultaneously applied in medicinal products as defined in the pharmaceutical law, which, as a matter of principle, is not applicable to dietary supplements. This is particularly noticeable in the group of products of botanical origin. The presence of such ingredients may cause problems within the scope of deciding whether a product is a dietary supplement or a drug. Such products and/or ingredients are referred to as “borderline” [2], and the popularity of such ingredients to a substantial degree depends upon consumer awareness within the scope of connections between diet and health [3]. Nevertheless, this is not the exclusively relevant factor. Even though fortified food (for instance, those fortified by means of the addition of vitamins) is not perceived by some consumers as food products that can improve the state of health, nevertheless, dietary supplements, due to their form, may be regarded as products having an actual influence upon the state of health more frequently than other fortified food products just like medicinal products [4].

The phenomenon of applying borderline ingredients in dietary supplements seems, therefore, to be an area requiring additional attention and regulations, and ensuring them may be problematic. Formally, dietary supplements are food products, and additional legal restrictions may have a negative influence on other aspects relevant to the food trade [5]. Moreover, determining certain legal frameworks rendering it possible to qualify, at least to a degree, the products discussed herein without additional consideration is not reflected in the decisions of the Court of Justice of the European Union, which adheres to the view that doubts relevant to the classification (food-pharma) in relation to borderline products ought to be resolved on a case-by-case basis [6,7].

Such an approach may, nevertheless, result in violating the rule of the free movement of goods by having the same product classified differently in different countries. This may be observed in the instance of melatonin, which is found to be a dietary supplement in France in the case of a dose of up to 2 mg, whereas in Poland, it is exclusively in the case of a dose not exceeding 1 mg; in turn, in the Czech Republic it is not legal to add it to dietary supplements [8].

To a certain degree, monitoring trade in dietary supplements that may have a different status in the particular member countries of the European Union (MS) is ensured by the Rapid Alert System for Food and Feed (RASFF) [8]. The role of the RASFF is to ensure the exchange of information on the presence of hazardous or illegal food products in the market between the member states. The data in this system, regardless of certain limits, may be useful within the scope of developing a food supervision policy; they also contribute to maintaining a high level of consumer awareness within the scope of existing hazards [9].

**Figure 1:**
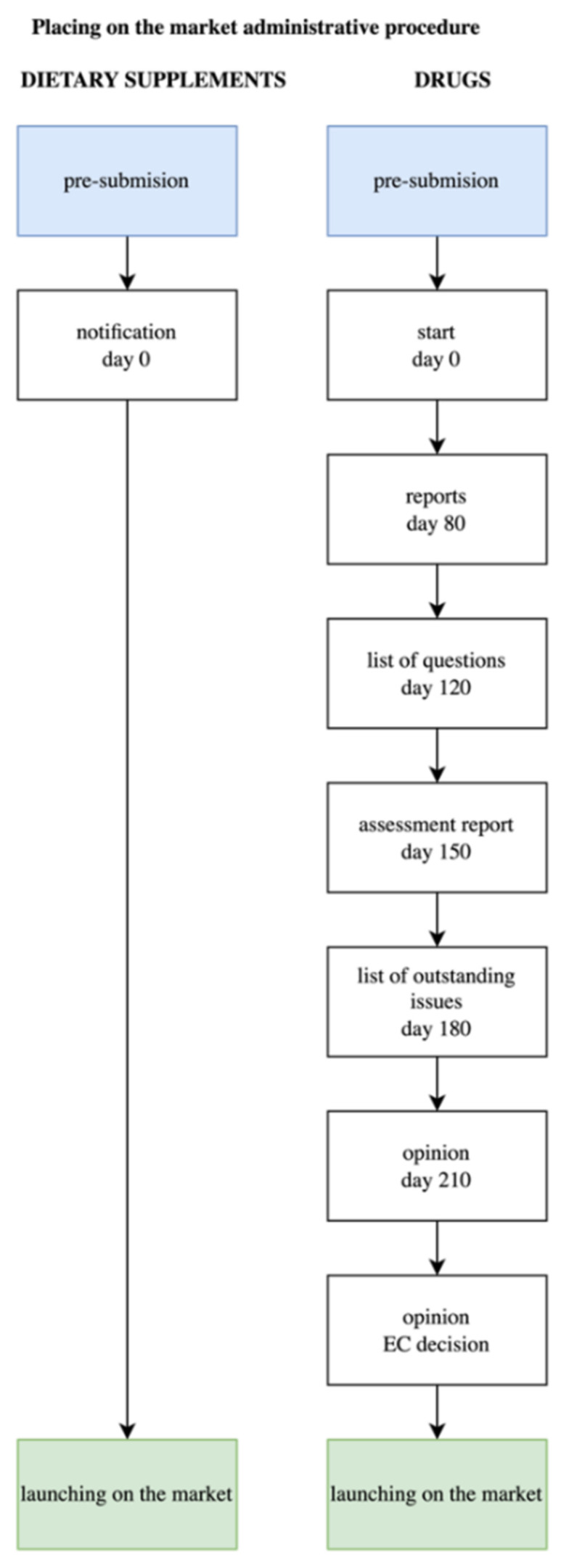
Scheme of the notification procedure of a dietary supplement in Poland in comparison with registering a medicinal product in the “common procedure” mode in the European Union, taking the relevant timeline under consideration.

In the context of the legislative discrepancies described herein, attention is worth paying to significant differences between dietary supplements and medicinal products to mention, but the process of registration and obtaining a marketing authorisation (Figure 1). The basic difference is that, as indicated by the name itself, the marketing authorisation (common procedure) of a medicinal product may render it marketable all over the EU and, therefore, be valid in the member states of the EU, even though it renders the procedure more time-consuming. Assessing an application relevant to a new drug may require up to 210 “active” days. This period of time is interrupted by at least a single “stop-clock”, during which an applicant prepares answers to the questions asked by the Committee for Medicinal Products for Human Use (CHMP). The clock is stopped after the 120th day, and another “stop-clock” is also possibly performed after the 180th day when the CHMP accepts a list of queries or unresolved issues, which ought to be seen by the company submitting an application. The assessment results issue an opinion by the CHMP by the 210th day. After that occurs, after 67 days, the European Commission usually makes a decision (issuing legally binding permission) [10].

Our earlier research led to the conclusion that the national register of functional foods, such as the RASFF, may be a valuable source of information in the process of the self-regulation of the market of dietary supplements in Poland; nevertheless, further research in relation to particular ingredients is worth undertaking. An analysis in that direction may help to identify dishonest market practices resorted to by the producers and distributors of non-conforming products [11]. This is of particular significance as we are observing an increase in dishonest market practices, the purpose of which is to generate more profits from the sale of fraudulent dietary supplements or misleadingly advertised [12] while their effective detection causes many difficulties, caused, among others, by lack of data on newly launched products on the market [13].

Because there is an extensive system of notification relevant to newly launched functional products in Poland, and that system concerns, in particular, dietary supplements, upon the basis of the data from this system, we conducted an analysis aiming to:

(1) Verify whether ingredients that may be conclusively classified as food or as medicinal products more frequently in comparison with other ingredients are entered into the national register as potentially non-conforming. Taking into consideration the form of researched products and also the resultant higher level of consumer trust in the aspect of its effectiveness in maintaining and improving the state of health, we presume that the presence of double-classified ingredients will predominantly be a reason for the CSI to initiate preliminary proceedings;
(2) Assess the participation of producers entered simultaneously in the national register of functional foods and also the national Register of Manufacturers and Importers of Medicinal Products, launching borderline ingredients as dietary supplements. We assumed that, as professional entities acquainted with the requirements of both of the markets (food-pharma), they will ensure that the food products launched by them will be relatively rarely found to be controversial or potentially non-conforming;
(3) Identify dishonest practices, the objective of which is to continue trading in products that are potentially non-conforming to the food law;
(4) Assess if there is a qualitative correlation between the causes of non-conformities in the national register and a category of notifications in the RASFF. We expect that due to a common system of notification and a clear procedure of reacting in the case of disclosing a potential non-conformity of a product is the subject of notification with the food law, the notifications relevant to exporting/importing products categorised in the RASFF as “Dietetic foods, food supplements and fortified foods”, “Composition” will be relatively rarely indicated as a hazard category.

### The Procedure for Notifying Dietary Supplements Newly Launched to the Market

In Poland, an entrepreneur wishing to launch a dietary supplement is obliged to notify the Chief Sanitary Inspector (CSI) of this intention with the use of electronic means of communication. An effective notification (i.e., signed by the authorised representative of an entity) renders it possible to commence the distribution and sale of a product the moment it is submitted. On the day following the notification being registered, data relevant to the product of which the Chief Sanitary Inspector has been notified are entered into the (national) electronic register of functional foods, and an entrepreneur receives an e-mail message informing them that a product has been entered into the register in question [14,15].

The Chief Sanitary Inspector′s prerogatives include the right to verify the quality of a product, in particular, in the aspect of its safety [14]. If the analysis of the submitted notification conducted by the Chief Sanitary Inspector does not give rise to doubts, an entrepreneur receives information that a product has been registered effectively. Nevertheless, if it is found that certain areas need a more profound analysis, preliminary proceedings can be initiated. These may take one of the two following courses, depending upon possible non-conformities. If a product′s non-conformity is unquestionable, for instance, if a product contains an ingredient that is not regarded as food whatsoever, the Chief Sanitary Inspector informs an entrepreneur and a competent state–county sanitary inspector (SCSI) in charge of the area on which an entrepreneur has their registered address. In the further course of the matters, an entrepreneur is obliged to withdraw a product conforming to an administrative decision issued by the state county sanitary inspector [16].

If it is found to be necessary to provide additional clarification on controversial issues, the Chief Sanitary Inspector also informs both the entrepreneur and a competent state–county sanitary inspector. In that case, the regional supervisory body issues a decision that trading in the product is to be temporarily suspended or, in extreme cases, that the product is recalled. In this latter case, an entrepreneur is obliged to take steps to collect the products in question from the entities with which it has contracts and which have purchased the product for the purpose of sales. In its decision, the Chief Sanitary Inspector itemises possible non-conformities and requests that a food market entity obtains an additional scientific assessment or present additional data [17].

**Figure 2:**
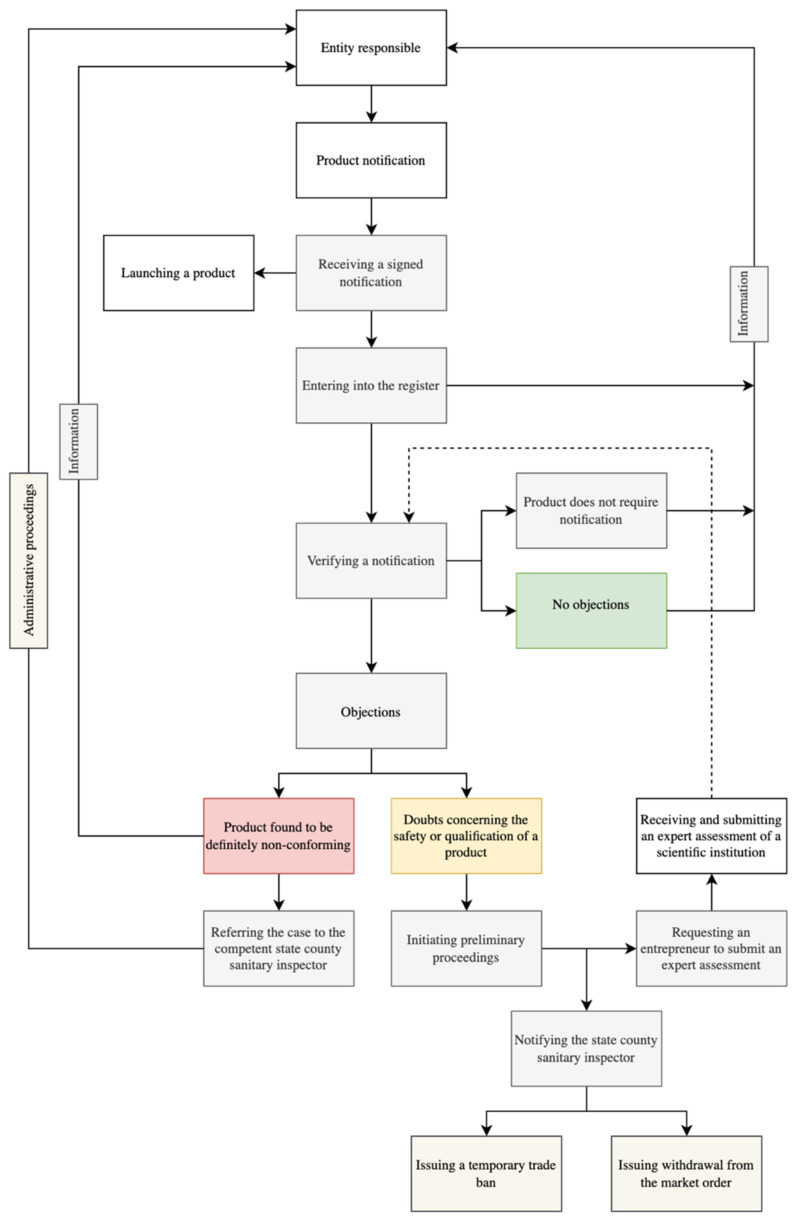
Scheme of a notification procedure applicable to intention to launch a functional product in Poland. The white boxes represent the actions of the reporting entity, the gray boxes are the actions of the Chief Sanitary Inspector, and the beige boxes are the actions of the state county sanitary inspector.

If an entrepreneur receives a positive assessment from a scientific institution or presents exhaustive additional documents, the Chief Sanitary Inspector ought to conclude the preliminary proceedings, notifying a competent state–county sanitary inspector, and that ought to result in a trade ban being repealed. Nevertheless, it is worth emphasising that the course of the procedures described herein is not set forth explicitly in the relevant regulations but rather constitutes a logical course of events in the light of the administrative law regulations in force in Poland [18]. It goes without saying that if a product is indeed found to be non-conforming and violating the food law regulations, a responsible entity ought to stop selling and distributing it [14]. The course of a notification procedure is presented in Figure 2.

## 2. Materials and Methods

### 2.1. Materials

#### 2.1.1. The National Register of Functional Foods

For the purpose of research, the national register of functional products for the period 5 February 2020–8 April 2022 was applied. The data for 2020 reflect the state as of 21 March 2021, whereas the data for 2021 (1 January 2021–28 November 2021) were collected on 28 November 2021. The data for the period 29 November 2021–8 April 2022 were acquired on 10 April 2022.

The data available in the register also cover an earlier period starting in 2007; nevertheless, in January 2020, an amendment was made to an ordinance [19], which actually entered into force in February. The amendment to the national regulation aimed to facilitate making notifications, but without substantive changes in the notification procedure itself. One of the changes was introducing drop-down lists of ingredients other than vitamins and minerals, including botanical ingredients, in the electronic notification form in place of the previous fields requiring the manual filling of the ingredient name. The above-mentioned change improved the quality of data in the register; in particular, it eliminated the double-entering of the same ingredient by registering it under synonymical or similar names (for instance, an extract of the seeds of milk thistle and an extract of milk thistle seeds).

#### 2.1.2. The Register of Medicinal Products and the National Register of Manufacturers and Importers of Medicinal Products

Substances qualified as medicinal were determined upon the basis of the register of medicinal products as of 24 March 2022, whereas entrepreneurs and importers of medicinal products were identified upon the basis of the National Register of Manufacturers and Importers of Medicinal Products (MIMP) as of 23 March 2022.

#### 2.1.3. RASFF

In order to achieve the objective of research within the scope of the RASFF, the data were acquired from it in the .csv format relevant to notifications in the system in the period 2020–2022. They were exclusively the data relevant to the group of “dietetic food, food supplements (…)” that were taken under consideration, and 671 notifications were thus identified. In the further course, their number was limited to those indicating Poland as a “notifying country” and/or “subject”.

### 2.2. Methods

#### 2.2.1. Selecting Products and Ingredients for Research

**Figure 3:**
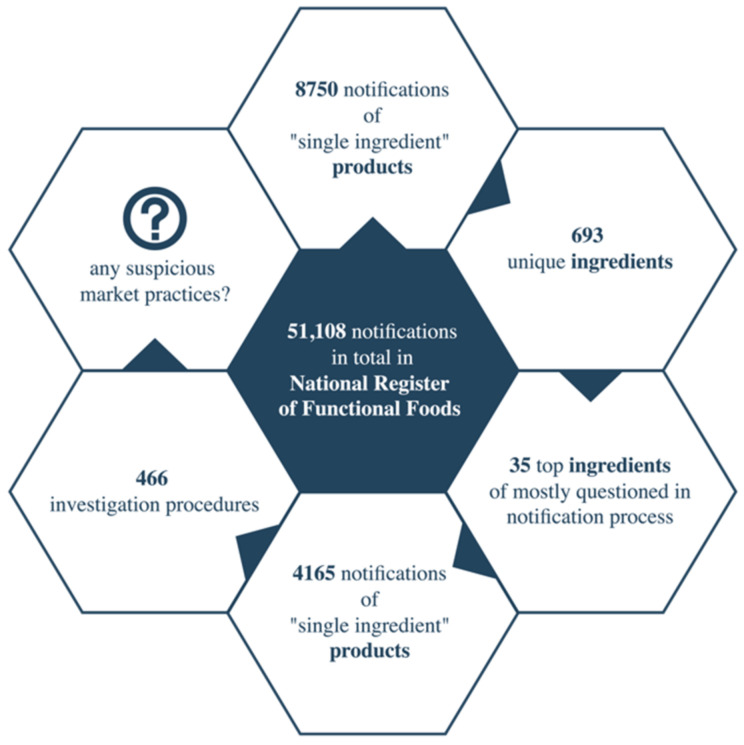
Scheme of input data for the analysis of the national registry.

Exclusively notifications relevant to products containing a single active ingredient hereinbelow referred to as “single-ingredient”, were selected (Figure 3).

It was ascertained that there were 8750 such products with 693 unique ingredients in the register in the analysed period of time. In the further course, those ingredients that had the highest potential non-conformity with the regulations of the food law applicable to functional food products were selected. For this purpose, the ABC method was applied; 5% of ingredients were arranged by a decreasing number of notifications reviewed and found to be potentially non-conforming and were qualified for further research. One of the following statuses was granted: “Does not meet the definition or requirements relevant to the proposed qualifications”, “The procedure was abandoned, the company withdrew from placing on the market”, “Proceedings Underway”, “Contains Not Permitted Ingredient”, “The product was abandoned on the market”.

For the purpose of further research, 35 ingredients in 4165 single-ingredient products, which were questioned by the competent authority in 466 cases altogether, were selected.

#### 2.2.2. Determining Borderline Ingredients

Each of the ingredients selected for analysis was assessed separately within the scope of being present simultaneously on the list of active substances marketable in Poland in the aspect of the pharmaceutical market. The assessment was conducted by means of searching the field “active substance” manually in the register of medicinal products (key: Table 1).

#### 2.2.3. Identifying Entities in Both of the Registers

In order to establish whether the analysed single-ingredient products are launched as well by entities in the market of trade in medicinal substances, the analysis was conducted with the use of the method of Text Mining and Text Miner in Statistica 13.1 (Statsoft). The methodology applied was identical to that described in the previous research of the authors [20]. The assessment also included the list of single-ingredient products acquired in accordance with the previous description. The criteria of analysis included: finding in the field “submitting entity” in the national register of the main parts of the name (i.e., for instance, the main part of the name for Pharmaucetial Company “A” Ltd. was A) of entities in the register of the producers and importers of medicinal products. In the first step, 229 entities from the register of the producers and importers of medicinal products were analysed. All research and scientific (16), veterinary (1), and health care entities (26) were excluded from the analysis. The entities that had a common, characteristic element of the name were also excluded, which, with the described method, made it impossible to automatically distinguish these entities in the national register (8).

#### 2.2.4. Assessment of the Activity of Entrepreneurs after Granting the Status of Potential Non-Conformity to the Notification

An assessment of the occurrence of the repeated submission of notification in the register was conducted. Exclusively entries to the register following earlier notification labelled as non-conforming and made by the same entity, the subject of which was certainly or highly likely the same product, were selected. The assessment of the similarity of a product was conducted upon the basis of a name and form of a product. Nevertheless, all the cases meeting the above-mentioned criterion, in which case the repeated notification containing information about the status indicated that the notification procedure was completed properly, were excluded. Such cases may indicate that the entity submitting a notification one more time rectified errors or controversies identified by the competent authority when the product was submitted for notification for the first time.

#### 2.2.5. Assessment of Compliance with the RASFF

Upon the basis of the descriptions of notifications in the field “subject”, a notification was generally classified in the aspect of the presence of a particular ingredient or substance, the presence of which is a product was a basis for entering it into the RASFF. It was verified whether a reason for notifying the RASFF is ingredients constituting the subject of research upon the basis of the national register. In the described method, only a manual comparative analysis was used.

## 3. Results

### 3.1. Borderline Ingredients

Permitted use in two functions was the case in relation to 25 ingredients: vitamin D, *Cannabis sativa* L., vitamin C, magnesium, melatonin, vitamin B12, cannabidiol (CBD), cannabidiol, carbon, vitamin E, dehydroepiandrosterone (DHEA), vitamin K, zinc, biotin, folic acid, tryptophan, potassium, vitamin A, niacin, propolis, fish oil, selenium, iron, *Camellia sinensis* (L.) Kuntze (tea plant), and riboflavin (vitamin B2).

Of all the notifications of single-ingredient products granted the status indicating doubts on the part of the food safety authority, 74% are relevant to so-called borderline ingredients. The borderline ingredients with the highest percent of notifications that may indicate non-conformities are vitamin D (17% of notifications with the status indicating uncertainty) and *Cannabis sativa* L. (10% of notifications).

### 3.2. The Participation of Producers and Importers of Medicinal Products

Of 35 ingredients, 11 were the subject of a notification by the entities in the National Register of Manufacturers and Importers of Medicinal Products (MIMP). Those were as follows: vitamin D (11% of all notifications), iron (11%), carbon (9%), fish oil (7%), zinc (7%), potassium (6%), vitamin C (5%), vitamin E (4%), biotin (3%), magnesium (2%) and vitamin B12 (1%).

Of the 466 analysed notifications, which were granted the status indicating doubts by the food safety authority, six were made by five different entities from the list of the MIMP. One of the six above-mentioned notifications was relevant to active carbon (as of the day of conducting research, the preliminary procedure is underway). The remaining five notifications concerned vitamin D—one of the entrepreneurs resigned from launching a product, and one was requested to provide additional data, whereas, in relation to two entrepreneurs, the proceedings were underway on the day of conducting our research. One of the entities in whose case the proceedings were underway made a preliminary notification of a product bearing a similar name (Table A1. Vitamin D: 12 December 2021 and 25 March 2021 on products—liposomal vitamin D 4000 IU) approximately 40 days after initiating the proceedings.

### 3.3. Comebacks Issue

Of the total number of 466 products that qualified for analysis and granted the status indicating non-conformity upon the basis of comparing their name and forms, it was ascertained in 76 cases that after initiating preliminary proceedings or refusing to register, notifying entity register a potentially identical product one more time. This kind of practice was applied by 42 entrepreneurs; therefore, 5% of all the entities (828) submitted notifications in the analysed period. The detailed data are presented in the Appendix A (Table A1).

The phenomenon in question was identified in relation to 24 of 35 selected ingredients, most frequently being products in capsules (35%), liquid forms (26%), in bulk (23%), whereas to the smallest degree, it was relevant to pills and dragees (14%).

In 33 cases, constituting nearly half of all the cases, resubmission was performed within 100 days after the initial submission, and the average time period between the first submission and resubmission was 51 days (median).

Notifications submitted within this period of time were most frequently relevant to liquid forms (15 cases) and capsules (12 cases).

Another period in which the most numerous resubmissions were observed was between 101 and 200 days after the first submission (average time: 148 days). Of 20 such submissions, eight were relevant to products in the form of powder, five in the form of capsules, four in liquid form, and three in the form of pills. The remaining 23 were submitted between the 201st and 300th day (on average, 270th day) after the first submission, and that was true for nine cases; four of them were relevant to products in the form of capsules, four in the form of pills and one the form of liquid, whereas in the period between 301st and 400th day (on average, 353rd day), eight products (namely seven capsules and one pill) were registered one more time. Between 401st and 700th day, six products were submitted for registration.

The products submitted the earliest were dietary supplements in the form of capsules and liquids. In the majority of cases, it was performed throughout the first 100 days. The products last to register one more time were dietary supplements in the form of pills; as it was observed, it was performed in the majority of cases between the 150th and 300th day.

Among the raw materials in relation to which more comeback was registered were *Cannabis sativa*, vitamin D, magnesium, carbon, vitamin B12, cannabidiol, and also Ganoderma lucidum. With the exception of carbon and *Cannabis sativa*, most of the comebacks were observed by the 160th day. Products containing carbon were registered one more time between 120th and 360th day, whereas those containing *Cannabis sativa* were even after 650th and 700th day (two products, one company).

The entities included in the research most frequently followed the practice described herein exclusively once (29 of 42 observations). Exclusively a few of the producers and/or distributors tended to use this practice of repeated submission more frequently, and that group includes those resubmitting a notification a few times in relation to a single raw material.

In relation to entities from the register of the MIMP, the practice of comeback was observed exclusively in the case of a single producer; nevertheless, it was applied as many as three times in relation to this same product containing vitamin D.

### 3.4. RASFF and National Register Connections

Analysing the RASFF led, in the initial phase, to selecting 78 submissions, constituting nearly 12% of all notifications in the group of “dietetic foods, food supplements and fortified foods”. The description of 20 of the selected notifications did not render it possible to conclusively determine the reason for finding a product to be non-conforming or hazardous. These submissions were verified by means of analysis from the level of “details” bookmark with the use of the RASFF Window. In the case of seven submissions, cause (s) was (were) established, whereas the 13 remaining were excluded from further analysis.

Twenty-six substances/groups of substances constituting the basis for entering a submission to the RASFF were identified; the detailed share of submissions is presented in the table below. Seven of the above-mentioned 26 groups were selected as relatively not frequent upon the basis of the national register (Table 2).

## 4. Discussion

The received results indicate that the ingredients which may be legally applied both in dietary supplements and medicinal substances (borderline ingredients) are frequently questioned in the process of notification; nevertheless, one ought to take into consideration that the reason for granting them the status of non-conforming products in the national register may be of a different kind. This non-conformity may result from too large of a dose of an ingredient, improper chemical form, health claims on a label that are not permitted, or formal errors in the notification submissions such as the lack of a signature of a person authorised to submit a notification. A large part of the selected borderline ingredients are popular and well-known vitamins and minerals, and also substances such as activated carbon melatonin.

The leading ingredients among those questioned single-ingredient products were vitamin D and also hemp derivatives which seem to be compatible with a lot of interest in these ingredients among consumers [21,22,23,24]. Both of the ingredients differ, nevertheless, in the aspect of the type of entities launching them. Whereas vitamin D in more than 10% of cases was offered by entities in the MIMP, hemp derivatives were applied exclusively by producers, not on the list of the MIMP. Interestingly, entities active simultaneously in the pharmaceutical product market launched exclusively supplements vitamins and minerals apart from carbon and fish oils. This group of ingredients is regulated relatively, not ambiguously, within the scope of the maximum permitted doses in dietary supplements. That would confirm the hypothesis that entities in the MIMP, being ex definitione acquainted with the industry-specific requirements and regulations, do not participate to a higher degree in trading substances to which few regulations apply in the aspect of the food law. Non-conformities and doubts were identified in the case of single-ingredient products distributed by this group of entities concerned, in most cases exclusively vitamin D.

The assessment of the conformity of notified submissions of the same entities gives rise to doubts about the degree to which a legislative procedure is applied to products in relation to which preliminary proceedings were initiated. Granting by a food safety authority of the status of potential non-conformity, in more than 16% of cases finished with repeated submission by this same entity, and presumably, the same product was submitted to be registered. That means that there is a sui generis interruption of a product lifecycle set forth in the regulation. The identified phenomenon leads to the situation in which the register contains the same product entered at least two times. Taking into consideration the scope of data provided for the national register and also the fact that central, regional, and local bodies of food supervision communicate exclusively in writing in the relevant scope (there is no central integrated register accessible to offices), it is possible that an entrepreneur launching a product, which was later questioned, recommences a submission procedure rather than disperses doubts by means of conducting appropriate additional research and submitting a declaration, etc., which may cause problems within the scope of identifying a product included in the preliminary proceeding while conducting control activities by local food supervision authorities. The possibility of abuse on the part of producers would be possible to reduce if a system permitted exchanging information on suspicious practices giving rise to concern effectively [25].

The phenomenon described herein also reveals, to a certain degree, factors rendering given products more risky as well as similar practices applied by various entities. We found that activities consisting of the repeated registration of the same product are resorted to by a relatively small number of producers—namely exclusively 5% of the researched companies. Nevertheless, it matters that this phenomenon was identified and described exclusively upon the basis of submitting single-ingredient products, which exclusively constitute more than 17% of all submissions in the analysed period. Regardless of limiting the sample to a specific group of products, we observed that repeated registration seems to depend upon the form of a dietary supplement. Moreover, it was two times more frequently seen in the case of capsules than pills. Powders and liquids were repeatedly submitted more frequently than pills, and this seems to be justified by the complexity of the technological process applied to the particular form and in the context of costs. The production of capsules and powders requires much less expense for investment within the scope of machines than that of pills [26]. Formulation rules are also simpler as [27]. The logical result is less serious consequences of, for instance, wrongly chosen proportions of ingredients, additional substances, and production parameters product; for instance, no lids are applied, and there are no broader problems with the compressed mass of which pills are made. Apart from the form of a product, it is worth indicating that the problem of repeated registration affects vitamins and minerals, excessively applied as supplements by the researched groups of the Polish population [28], and even though vitamins in the form of dietary supplements ought to be safer than their counterparts in medicinal products applied for particular therapeutic purposes it is disturbing that the phenomena described herein by us are seen as well in the case of ingredients with a high potential of negative effects, such as vitamins A, E, and D [29].

Another factor in which case certain trends can be seen is the time after a product is resubmitted. Most of such steps were taken after, on average, approximately 50–150 days. This is an interesting result in the context of the audit of the Supreme Audit Office (SAO) in 2017, in which a long (exceeding 455 days) average time of processing submission notifications [30,31]. The result found in our research might prove that the reaction time of the CSI was reduced a lot.

What differs from the time periods described herein is what was seen in the case of submissions relevant to active carbon, in which case repeated notification was made no sooner than 130 days after the first submissions. It is worth emphasising that in the light of Commission Regulation (EU) No 432/2012 of 16 May 2012, establishing a list of permitted health claims made on foods [32], also applicable to dietary supplements, consuming active carbon in the daily dose of 1 g helps to reduce excessive stomach bloating after a meal. Simultaneously, a medicinal product containing active carbon (in the dose of 200 mg) is registered, which illustrates the reality of borderline ingredients well.

The market presence of borderline products non-conforming to the food law requirements or violating the pharmaceutical law, contrary to the hypothesis formulated at the beginning of this article, was confirmed in the entries into the RASFF. The conducted analysis permitted identifying a dozen or so submissions relevant to seven ingredients found by us to be borderline. It may mean that, regardless of the simple and cost-free procedure of notification, entrepreneurs fail to comply with the duty of submitting the notification of trading in dietary supplements. Such a hypothesis is backed up by the presence of a large number of submissions in the RASFF relevant to the presence of yohimbine, regardless of a noticeable fall in notification submissions for products containing it observed since the beginning of 2019 [11]. Under the RASFF, it was also found that there are typically medicinal substances that cannot be entered into a notification form. That refers to substances referred to as “SARMs”, which are a class of anabolic agents. The presence of such products in supplements is a problem known and monitored (to a substantial degree) [33]. This issue is beyond the scope of our research.

The presence in Poland of the register of dietary supplements, which was the basis for our research, provides broad possibilities for analysing trends and phenomena. As was shown by research, this tool has a lot of potentials and may be widely applied if developed by various participants such as state and scientific institutions and businesses [34].

## 5. Conclusions

As a result of the analyses, the following relationships were formulated:

1. Member States’ regulations (at the level of national law) may be unable to monitor borderline products effectively. It is recommended to use regulations at the level of the EU;
2. The repeated notification of the same product is most frequent among pharmaceutical products that do not require highly specialised equipment and technology to be produced—liquids, powders, and capsules;
3. Repeated submission is most frequently seen within the first 50–100 days—thematic control of the SCSI ought to, therefore, be conducted right then;
4. Producers from the same drug producers registering dietary supplements containing borderline ingredients, nevertheless, were not found to resort to “comeback” in relation to borderline ingredients;
5. Partial automatising of the national register has reduced the processing time of notifications by the CSI. Its further development is recommended to strengthen supervision over public health. It is advisable to regulate legal matters of striking out the withdrawn/recalled products of the national register;
6. Research into multi-ingredient products ought to be continued.

## Figures and Tables

**Table 1:** Specifications of words upon the basis of which ingredients were classified as borderline.

National Register	Register of Medicinal Products
Vitamin D	Cholecalciferolum/Vitaminum D3
*Cannabis sativa* L.	Cannabidiolum
Vitamin C	Acidum ascorbicum
Magnesium	Magnesium
Melatonin	Melatoninum
Vitamin B12	Cyanocobalaminum
Cannabidiol (CBD)	Cannabidiolum
Cannabidiol	Cannabidiolum
Carbon	Carbo activatus
Vitamin E	Tocofersolanum/int-rac-alfa-Tocopherylis acetas
Dehydroepiandrosterone (DHEA)	Dehydroepiandrosteronum
Coenzyme Q10	n/a
Vitamin K	Phytomenadionum
Protein	n/a
Zinc	Zinci oxidum/Zinci sulphas/Zinci chloridum
Biotin	Biotinum
Pinus massoniana Lamb (Masson′s pine)	n/a
Folic acid	Acidum folicum
Tryptophan	Teleniteum
Azadirachta indica (Neem)	n/a
Creatinine	n/a
Potassium	Potassium hydrogen carbonate/Potassium citrate
Vitamin A	Retinoli palmitas/Retinoli acetas
Niacin	Nicotynamidum
Propolis	Propolis extractum
Fish oil	Fish oil, rich in omega-3-acids
Methylosulfonylomethane (MSM)	n/a
Selenium	Sodium selenite
Phitocannabinoids	n/a
Iron	Ferrosi sulfas/Ferrum (III)
*Ganoderma lucidum* (Curtis) P.Karst.	n/a
*Camellia sinensis* (L.) Kuntze (tea plant)	Cameliae sinensin folii extractum siccum
*Mucuna pruriens* (L.) DC. (monkey tamarind)	n/a
Riboflavin (Vitamin B2)	Riboflavinum (vitamin B2)
Colagen	n/a

**Table 2:** List of ingredients/group of ingredients constituting the basis to enter a product into the RASFF in the group of “dietetical food (…)”.

Ingredient/Group of Ingredients	Total Count of Each Ingredient/Group of Ingredients in the RASFF
Yohimbine	20
SARMs (Selective Androgen Receptor Modulators)	10
Hemp derivatives *	*7*
DMAA (1,3-dimethylamylamine)	6
Ephedra	5
Medicinal ingredients, L-threonate	3
Hupercine, Vitamin B12 *, Caffeine	2
Piperine, Phenylethylamine, Beta-alanine, *Hydrastis canadensis* L. (orangeroot), Folic acid *, Conezyme Q10 *, Heavy metals, Vitamin E *, 5-HTP (5-hydroxytryptophan), Ethylene oxide, Melatonin *, Methyl synephrine, Mucuna pruriens *, Synephrine, Mixed composition of botanicals, DMAE (Dimethylethanolamine)	1

* Items in italics simultaneously identified as potentially most frequently non-conforming upon the basis of the national register.

## Data Availability

Publicly available datasets were analysed in this study. These data can be found here: https://powiadomienia.gis.gov.pl (accessed on 21 March 2021, 28 November 2021, 10 April 2022); https://www.gov.pl/web/gif/rejestry-wytworcow-importerow-i-dystrybutorow (accessed on 27 March 2022); https://rejestrymedyczne.ezdrowie.gov.pl/registry/rpl (accessed on 24 March 2022); https://data.europa.eu/data/datasets/restored_rasff?locale=en (accessed on 11 April 2022).

## Appendix A

**Table A1:** Re-notifications of notifications of potentially the same products.

Active Ingredient	Case Number	Date	Product Name	Form	Product Category	Company Number	Status	The Number of Days between the First and the Next Notification
Melatonin	1	26 February 2021	********* ***** Melatonin 3 mg—120 tablets	tablets	DS	4	PP	206
		20 September 2021	********* ***** Melatonin 3 mg—240 tablets	tablets	DS	4		
	2	19 February 2020	Melatonin Forte+	tablets	DS	39	PA-WITH.	258
		3 November 2020	Melatonin 5 mg	tablets	DS	39	PP	
*C. sativa* ^(1)^	3	15 May 2020	********* 5%	Liquid (drops)	DS	7	PP	664
		10 March 2022	5% Broad Spectrum hemp oil	Liquid (drops)	DS	7		
	4	15 May 2020	********* 10%	Liquid (drops)	DS	7	PP	664
		10 March 2022	10% Broad Spectrum hemp oil	Liquid (drops)	DS	7		
	5	22 January 2021	CBD Oil 5%	Liquid (drops)	DS	24	PA	80
		12 April 2021	Hemp Oil Full Spectrum 5% Hemp Oil	Liquid (oil)	DS	24		
	6	22 January 2021	CBD Oil 10%	Liquid (drops)	DS	24	PA	80
		12 April 2021	Hemp Oil Full Spectrum 10% Hemp Oil	Liquid (oil)	DS	24		
	7	22 January 2021	CBD Oil 15%	Liquid (drops)	DS	24	PA	80
		12 April 2021	Hemp Oil Full Spectrum 15% Hemp Oil	Liquid (oil)	DS	24		
Vitamin D	8	28 October 2020	********* 4000	capsules (soft capsules)	DS	1	PA-WITH.	307
		31 August 2021	********* 4000	capsules	DS	1		
	9	12 January 2021	********** Vitamin D3 10,000 60 caps	capsules	DS	4	PP	353
		31 December 2021	********** VITAMIN D3 10,000 120 gelcaps	capsules	DS	3		
	10	26 February 2021	********* ***** Vitamin D3 5000 IU—100 capsules	capsules	DS	4	PP	68
		5 May 2021	********* ***** Vitamin D3 5000 IU—200 capsules	capsules	DS	4		
	11	23 July 2021	********* ***** Vitamin D3 10,000 IU—100 capsules	capsules	DS	4	PP	174
		13 January 2022	********* ***** Vitamin D3—100 capsules	capsules	DS	4	PP	
	12	23 December 2021	Vitamin D3	Liquid (spray)	DS	5	PA	63
		24 February 2022	Vitamin D3	Liquid (spray)	DS	5		
	13	20 May 2020	****** D 100 µg	capsules	DS	6	PP	365
		20 May 2021	****** D 100 µg	capsules (soft capsules)	DS	6		
	14	18 May 2020	Vitamin D-3 2000 IU	capsules	DS	18	NTBC	17
		4 June 2020	Vitamin D3 2000 IU	capsules	DS	18		
	15	2 June 2021	Vitamin D3—Highest Potency	capsules	DS	18	PP	55
		27 July 2021	Vitamin D3—Highest Potency	capsules	DS	18		
	16	12 February 2021	********** *** Liposomal Vitamin D 4000 IU	powder (sachets)	FSMP	21 (MIMP list)	PP	41
		25 March 2021	********** Liposomal Vitamin D 4000 IU	powder (sachets)	DS	21 (MIMP list)	PA	
	17	5 May 2020	Vitamin D3 5000 IU	capsules	DS	22	PP	175
		27 October 2020	Vitamin D3	capsules	DS	22	NTBC	
	18	22 July 2020	Vitamin D in drops	Liquid (drops)	DS	28	PA-WITH.	61
		21 September 2020	Vitamin D in drops	Liquid (drops)	DS	28		
	19	28 October 2020	******* ******* Vitamin D3 10,000 360k from Lanolin	capsules	DS	29	PP	301
		25 August 2021	******* ******* Vitamin D3 10,000 IU, 360 Softgels	capsules	DS	29	PP	
	20	30 October 2020	******* ******* Vitamin D3 10,000 IU 120 capsules	capsules	DS	29	PP	299
		25 August 2021	******* ******* Vitamin D3 10,000, 120 Softgels	capsules	DS	29	PP	
	21	9 June 2021	Vitamin D3, 5000 IU	capsules	DS	30	PP	28
		7 July 2021	Vitamin D3, 5000 IU	capsules	DS	30		
	22	28 July 2020	***** * ****	Liquid (drops)	IF	32	NMD	189
		2 February 2021	***** * ****	Liquid (drops)	DS	32		
	23	10 April 2020	d3 5000	capsules	DS	34	PP	467
		21 July 2021	****** D3 5000 iu 240 VC	capsules	DS	34		
	24	10 June 2020	****** vitamin D3 4000 IU for obese adults	capsules (soft capsules)	DS	40	PP	349
		25 May 2021	****** vitamin D3 4000 IU for healthy people over 75 years of age	capsules (soft capsules)	DS	40		
	25	17 March 2020	Vitamin D-3 5000 IU	tablets	DS	42	PA-WITH.	391
		12 April 2021	*** VITAMIN D3 5000 IU	tablets	DS	42		
DHEA ^(2)^	26	26 February 2021	********* ***** DHEA 50 mg—50 tablets	tablets	DS	4	NPI	270
		23 November 2021	********* ***** Dehydroepiandrosterone—50 tablets	tablets	DS	4		
	27	27 April 2021	******* DHEA 50 mg—120 capsules	capsules	DS	4	NPI	87
		23 July 2021	******* DHEA 50 mg—120 capsules	capsules	DS	4	NPI	
	28	27 April 2021	******* DHEA 25 mg—120 capsules	capsules	DS	4	NPI	66
		2 July 2021	******* DHEA 25 mg—30 capsules	capsules	DS	4	NPI	
*P. massoniana* ^(3)^	29	30 April 2021	Pine pollen—50 g	powder	DS	26	PA	14
		14 May 2021	Pine pollen—50 g	powder	DS	26	PA	
	30	30 April 2021	Pine pollen—100 g	powder	DS	26	PA	14
		14 May 2021	Pine pollen—100 g	powder	DS	26	PA	
Selenium	31	15 May 2020	Selenium 200 mcg—100 tablets	tablets	DS	4	NTBC	584
		20 December 2021	****** Selenium 200 mcg—100 tablets	tablets	DS	4		
*C. sinensis* ^(4)^	32	5 November 2020	Organic Green Tea **** ****	capsules	FSMP	14	NTBC	194
		18 May 2021	Organic Green Tea **** ****	capsules	DS	14		
Magnesium	33	15 February 2022	**** ********* Magnesium Citrate 100 mg—100 capsules	capsules	DS	4	PA	49
		5 April 2022	**** ********* Magnesium Citrate 100 mg—100 capsules	capsules	DS	4		
	34	18 May 2020	Magnesium Citrate caps.	capsules	DS	18	NTBC	17
		4 June 2020	Magnesium Citrate caps	capsules	DS	18		
	35	4 June 2020	Magnesium Chloride Hexahydrate	powder	DS	27	PA	111
		23 September 2020	Magnesium Chloride Hexahydrate	powder	DS	27		
	36	9 August 2021	*** ***** Magnesium citrate pure power	powder	DS	35	PA	14
		23 August 2021	*** ***** Magnesium citrate pure powder	powder	DS	35		
*M. pruriens* ^(5)^	37	17 June 2021	L-DOPA 300 mg Scabies extract	capsules	DS	37	PP	263
		7 March 2022	L-DOPA 300 mg Scabies extract	capsules	DS	37		
Coal	38	21 February 2020	Carbon C60	capsules	DS	16	PA	353
		8 February 2021	Active carbon	capsules (vegetarian capsules)	DS	16		
	39	20 March 2020	Activated carbon	powder (granules)	DS	26	PA-WITH.	139
		6 August 2020	Activated carbon	powder	DS	26	PA-WITH.	
	40	22 March 2021	Activated carbon powder	powder	DS	36	PA-WITH.	130
		30 July 2021	Activated carbon 100 g	powder	DS	36	PA	
	41	21 April 2020	Active carbon	powder	DS	44	PA-WITH.	191
		29 October 2020	Activated carbon	powder	DS	44	PP	
Zinc	42	13 April 2021	Zinc, 50 mg—100 tablets	tablets	DS	30	PP	125
		16 August 2021	***** Zinc Picolinate, 50 mg	capsules (hard capsules)	DS	30	NTBC	
Biotin	43	15 April 2020	Biotin 10,000 mcg	tablets	DS	18	PP	50
		4 June 2020	Biotin 10,000 mcg	capsules	DS	18	PP	
	44	18 May 2020	Biotin	capsules	DS	18	PP	438
		30 July 2021	Biotin	capsules	DS	18		
Fish oil	45	5 March 2020	********* ******* 3 types of fish oils	Liquid (oil)	FSMP	23	PA	215
		06 October 2020	**************** Immuno and Neuro Lipids	Liquid (oil)	FSMP	23	PP	
Creatine	46	18 May 2020	**** Creatine	powder	DS	10	PA	686
		4 April 2022	**** Creatine 1.0	powder	DS	10		
	47	29 December 2021	************ Creatine	capsules	DS	42	NTBC	47
		14 February 2022	************ Creatine	capsules	DS	42		
Vitamin B12	48	17 September 2020	B12 Hydroxocobalamin	tablets	DS	20	PP	180
		16 March 2021	B12 Hydroxocobalamin 500 µg	tablets	DS	20		
	49	29 January 2021	VIT. B12 1000	capsules	DS	38	PA-WITH.	164
		12 July 2021	VIT. B12 1000	tablets	DS	38	PP	
	50	23 April 2020	******** Vitamin B12 900 mcg	capsules (hard capsules)	DS	25	PP	382
		10 May 2021	******** Vitamin B12 950 µg	capsules	DS	25		
	51	8 June 2020	***** *** Vitamin B12 with strawberry flavor	tablets (lozenges)	DS	31	PA-WITH.	38
		16 July 2020	***** *** vitamin B12 with strawberry flavor	tablets (lozenges)	DS	31		
Vitamin E	52	28 July 2021	Vitamin E 400 iu 90 capsules	capsules (soft capsules)	DS	8	PP	134
		9 December 2021	Vitamin E 400 IU 90 capsules	capsules (soft capsules)	DS	8	PA	
	53	23 February 2021	Vitamin E-400 With Mixed Tocopherols 100 Softgels	capsules	DS	18	PP	3
		26 February 2021	Vitamin E Mixed Tocopherols 100 Softgels	capsules	DS	18		
	54	23 February 2021	Vitamin E-400 With Mixed Tocopherols 250 Softgels	capsules	DS	18	PP	3
		26 February 2021	Vitamin E Mixed Tocopherols 250 Softgels	capsules	DS	18		
Vitamin K	55	22 January 2021	****** VITAMIN K2 MK-7 200MCG 250TAB	tablets	DS	15	NTBC	276
		25 October 2021	****** VITAMIN HIGH STRENGTH VITAMIN K2 MK-7 200MCG 250KAPS	capsules	DS	15		
	56	22 January 2021	****** VITAMIN K2 MK-7 200MCG 100TAB	tablets	DS	15	NTBC	276
		25 October 2021	****** VITAMIN HIGH STRENGTH VITAMIN K2 MK-7 200MCG 100KAPS	capsules	DS	15		
Vitamin A	57	29 April 2020	****** Vitamin Vitamin A 1000iu 250tab	tablets	DS	15	PP	285
		8 February 2021	****** VITAMINA VITAMIN A 250TAB	tablets	DS	15		
Cannabidiol	58	31 March 2020	5% RAW OIL HempOil 500 mg CBD + CBDa	Liquid (oil)	DS	9	PA-WITH.	93
		2 July 2020	HempOil 500 mg	Liquid (oil)	DS	9	PP	
	59	31 March 2020	10% RAW OIL HempOil 1000 mg CBD + CBDa	Liquid (oil)	DS	9	PA-WITH.	93
		2 July 2020	HempOil 1000 mg	Liquid (oil)	DS	9	PP	
	60	31dt March 2020	15% RAW OIL HempOil 1500 mg CBD + CBDa	Liquid (oil)	DS	9	PA-WITH.	93
		2 July 2020	HempOil 1500 mg	Liquid (oil)	DS	9	PP	
	61	31 March 2020	30% RAW OIL HempOil 3000 mg CBD + CBDa	Liquid (oil)	DS	9	PA-WITH.	93
		2 July 2020	HempOil 3000 mg	Liquid (oil)	DS	9	PP	
Cannabidiol (CBD)	62	21 June 2021	CBD Oil 5%, 500 mg, Full spectrum	Liquid (drops)	DS	2	PA	72
		1 September 2021	CBD Oil 5%, 500 mg, Full spectrum	Liquid (drops)	DS	2		
	63	21 June 2021	CBD Oil 10%, 1000 mg, Full spectrum	Liquid (drops)	DS	2	PA	72
		1 September 2021	CBD Oil 10%, 1000 mg, Full spectrum	Liquid (drops)	DS	2		
	64	17 August 2021	CBD Oil 15%, 1500 mg, Full spectrum	Liquid (drops)	DS	2	PA	15
		1 September 2021	CBD Oil 15%, 1500 mg, Full spectrum	Liquid (drops)	DS	2		
	65	2 September 2021	CBD oil 500 mg, concentration 5%	Liquid (oil)	DS	17	PP	145
		25 January 2022	CBD oil 500 mg, concentration 5%	Liquid (oil)	DS	17		
	66	2 September 2021	CBD oil 1000 mg, concentration 10%	Liquid (oil)	DS	17	PP	145
		25 January 2022	CBD oil 1000 mg, concentration 10%	Liquid (oil)	DS	17		
	67	2 September 2021	CBD oil 2000 mg, concentration 20%	Liquid (oil)	DS	17	PP	145
		25 January 2022	CBD oil 2000 mg, concentration 20%	Liquid (oil)	DS	17		
MSM ^(6)^	68	6 December 2021	MSM ORGANIC SULFUR	powder	DS	11	NTBC	108
		24 March 2022	MSM ORGANIC SULFUR	powder	DS	11		
*G. lucidum* ^(7)^	69	30 April 2021	Reishi	tablets	DS	26	PA	152
		29 September 2021	Reishi	tablets	DS	26		
	70	30 April 2021	REISHI powder—50 g	powder	DS	26	PA	152
		29 September 2021	REISHI powder—50 g	powder	DS	26		
	71	30 April 2021	REISHI powder—100 g	powder	DS	26	PA	152
		29 September 2021	REISHI powder—100 g	powder	DS	26		
Phytocannabinoids	72	10 November 2021	Oil 10% hemp macerate	Liquid (oil)	DS	12	PP	35
		15 December 2021	10% phytocannabinoids macerate	Liquid (oil)	DS	12		
	73	10 November 2021	Macerate Oil 15% hemp macerate 10% phytocannabinoids	Liquid (oil)	DS	12	PP	35
		15 December 2021	Macerate of 15% phytocannabinoids	Liquid (oil)	DS	12		
	74	14 April 2021	** ****** Premium Hemp Oil Decarbo 5%	Liquid	DS	33	PA	12
		26 April 2021	******** Premium Hemp Oil Decarbo 5%	Liquid (drops)	DS	33		
Protein	75	25 August 2020	******** ***	powder	FSMP	43	NMD	125
		28 December 2020	************	powder	FSMP	43	PP	
	76	12 May 2020	******* * WPC 82	powder	DS	19	PA	99
		19 August 2020	******** WPC 82	powder	DS	19	PA	

DS—Dietary Supplement; FSMP—Food for Special Medical Purposes; IF—Infant Foods; PP—Proceedings Underway; PA-WITH.—The procedure was abandoned, the company withdrew from placing on the market; PA—The product was abandoned on the market; NTBC—The notification needs to be completed; NMD—Does not meet the definition or requirements for the proposed qualification; NPI—Contains Not Permitted Ingredient. ^(1)^ *Cannabis sativa* L. (*Cannabis sativa*), ^(2)^ dehydroepiandrosterone (DHEA), ^(3)^ Pinus Massoniana Lamb. (Masson’s Pine), ^(4)^ *Camellia sinensis* (L.) Kuntze (Chinese Tea), ^(5)^ *Mucuna pruriens* (L.) DC. (Scabies), ^(6)^ methylsulfonylmethane (MSM), ^(7)^ *Ganoderma Lucidum* (Curtis) P. Karst. (Yellowish lacquer). * The names of products and entrepreneurs have been anonymised using the symbols “*”.
